# Toxicity, Safety, and Efficacy Studies on Mesenchymal Stem Cells Derived from *Decidua basalis* in Wistar Albino Rats by Intravenous and Subcutaneous Routes

**DOI:** 10.3390/cimb44090277

**Published:** 2022-09-06

**Authors:** Priya Subramani, Jaianand Kannaiyan, Saurabh Khare, Paulraj Balaji, Atif Abdulwahab A. Oyouni, Saad Ali S. Aljohani, Mishal Olayan Alsulami, Osama M. Al-Amer, Othman R. Alzahrani, Malik A. Altayar, Afrah Awadh Allah Alsulami, Veeramanikandan Veeramani

**Affiliations:** 1 PG and Research Centre in Microbiology, MGR College, Hosur 635109, India; 2 Research and Development, CellCure Therapeutics, Coimbatore 641046, India; 3 Research and Development, Dr. Scientific Solutions, Gurgaon 122003, India; 4 PG and Research Centre in Biotechnology, MGR College, Hosur 635109, India; 5 Department of Biology, Faculty of Sciences, University of Tabuk, Tabuk 71491, Saudi Arabia; 6 Genome and Biotechnology Unit, Faculty of Sciences, University of Tabuk, Tabuk 71491, Saudi Arabia; 7 Department of Clinical Pharmacy, Faculty of Health Sciences and Nursing, Alrayan Colleges, Almadinah 42541, Saudi Arabia; 8 Cytogenetic and Molecular Genetics, Clinical Laboratory Department, Prince Sultan Medical City, Riyadh 11159, Saudi Arabia; 9 Department of Medical Laboratory Technology, Faculty of Applied Medical Sciences, University of Tabuk, Tabuk 71491, Saudi Arabia; 10 Ministry of Environment, Water, and Agriculture, Riyadh 11195, Saudi Arabia

**Keywords:** *decidua basalis*, mesenchymal stem cells, maximal tolerance dose, minimal lethal dose, lethal dose

## Abstract

*Ex vivo* expanded decidua-basalis(DB)-derived mesenchymal stem cells (MSCs) obtained from single donors have demonstrated therapeutic benefits in in vitro and in vivo studies. In this report, the intravenous and subcutaneous administration of DB-MSCs obtained from five healthy donors was assessed considering clinical grade proliferation, accessibility, and toxic effects in Wistar albino rats. The ability of the obtained DB-MSCs for differentiating, as well as their expression of several cell surface markers and immunomodulatory activities, were all assessed. Clinical standard proliferated cells were administered to animals intravenously and subcutaneously in a series of preclinical models in order to assess their in vivo toxicity, general safety, and tumorigenic possibilities. We established that DB cells exhibit structural and functional traits with MSCs. At various doses supplied intravenously or subcutaneously, the research showed no fatality, abnormal response to therapy, or substantial pathological modifications in the rats. Furthermore, there was no indication of prenatal damage in the same animal species when the rats were repeatedly treated with DBMSCs. Thus, DBMSCs were demonstrated to be non-toxic, non-teratogenic, and non-tumorigenic. To determine whether they can be administrated to human patients without risk, more investigation is recommended.

## 1. Introduction

A number of methods are currently being developed to construct various cell-based products as candidates for pharmacological assessment. Among the various cell therapy products, MSCs are being widely researched in order to comprehend their therapeutic benefits. Nevertheless, further research is needed to identify various clinical constraints and the practicability of using MSCs. Another important problem encountered in the treatment process is the lack of clarity on the auto-renewal of decidua-basalis-derived mesenchymal stem cells (DB-MSCs) embedded on the target site for a specific time period. Further complications arise due to the obscurity about their in vivo multilineage differentiation potential. As the number of DB-MSCs required for tissue regeneration is so large, scaling up is required for reliable therapeutic application [1,2,3,4].

As a result, it is critical to determine the conditions for good manufacturing practices for pharmaceutical manufacture, minimal dosages mandatory for minimizing thrombo-embolic consequences, and possible contaminations. Considering the benefits and drawbacks of embryonic stem cells, a large number of clinical and preclinical investigations have advised using adult MSCs for cell-based reparative therapy approaches [5,6]. However, because of the laborious process of isolation and the unsuitability of MSCs for allogeneic transplants, as well as harmfulness of MSCs derived from adipose tissues or bone marrow for clinical usage [7,8], their applications are limited in cell-based therapy. DB-MSCs are of great interest for researchers as they are easily accessible, ethically safe, and tolerant to immunological effects. Another key factor is their isolable homogeneous population in order to meet existing market needs [9,10].

The objective of this proposed study is to investigate the complete characterization, the up-scaling of cells derived from *decidua basalis*, and their efficacy upon administration through intravenous and subcutaneous injection in Wistar albino rats. This animal model study will provide insight into the suitability of DB-MSCs for clinical therapy and an impetus for further research.

## 2. Materials and Methods

### 2.1. In Vitro Study

#### Material Source

*Decidua basalis* (n = 5), the maternal portion of the placenta, was obtained through a caesarean section performed with informed consent and the entire study was duly approved by the institutional ethical committee. The work followed established cGLP and cGMP criteria and all of the tissue culture procedures were carried out in a cleanroom class 1000 environment. The *decidua basalis* region of the placenta was used to isolate MSCs and was expanded up to the tenth passage in the laboratory, under the Class II Type A2 biosafety cabinet, and their characteristics were assessed by the proliferation rate, phenotypic characterization using fluorescence, genotypic characterization using karyotyping, and cytochemical staining for mesodermal differentiation. The methodologies for the isolation, expansion, and characterization of DBMSCs were adopted from previous reports [1,2,11,12] (Jaianand et al., 2018; Jaianand et al., 2017; Jaianand and Balaji, 2015; Jaianand and Balaji, 2015).

### 2.2. In Vivo Study

#### Experimental Animals

The Institutional Animal Ethics Committee (IAEC) and Institute for Toxicological Investigations endorsed the study design and animal allocation (Nandha Pharmacy). Briefly, samples of five different batches of MSCs were administered Wistar albino rats subdivided into five groups, with five animals in each sex. Group 1 animals acted as the control group. Group 2 and Group 3 animals were given an intravenous shot, while Group 4 and Group 5 animals were given a single subcutaneous injection at a dose of 10 × 10^6^ MSCs/kg body weight, which is ten times the maximum human therapeutic dose. In the vehicle control group, 0.9% NaCl_2_ injection was used as a diluent. The body weights of all of the animals were measured before treatment (day 1) and regularly afterward. After 14 days, they were monitored for death and indications of toxicity. Upon completion of the study, all rats were euthanized and exposed to a thorough necropsy. All of the tests were carried out in accordance with CPCSEA criteria.

### 2.3. Administration

Each rat received a single bolus of the dispersion via slow intravenous injection in the lateral tail vein and subcutaneous injection in the flank area. The shots were administered using a sterile hypodermic syringe and a stainless-steel needle (26 G). The dosage was 10 mL/kg body weight and was adjusted based on the animal’s body weight on the day of treatment.

### 2.4. Mortality and Clinical Signs

The experiments were conducted on rats of both sexes along with a control group. During the first four hours following injection, all animals were monitored for mortality (at intervals of 30 min, 1 h, 2 h, and 4 h), and then once a day for the next 14 days. Any cases of death that occurred during the study period were documented.

In the same way, all animals were monitored for indications of toxicity (at intervals of 30 min, 1 h, 2 h, and 4 h), and then once a day for the next 14 days. The presence of any symptoms, progression, and disappearance, if any, was documented.

### 2.5. Body Weight

The body weights were measured the day before treatment, and on days 7 and 15. The change in body weight of the individual animals with respect to the initial measurement and group mean values were calculated.

### 2.6. Necropsy

After the study period, on day 15, the animals were sacrificed by CO_2_ asphyxiation and subjected to necropsy. The gross pathological changes were documented by examination under a microscope.

### 2.7. Safety Study

In the safety investigation, both genders of Wistar albino rats (180–200 gm) were examined. The vehicle (1 mL saline/kg) was delivered intravenously to Group 1 as a control. The animals belonging to Group 2–5 were administered only with MSCs. The test animals were injected intravenously once (1 × 10^7^ cells/head/body/kg weight). The rats were monitored for 13 weeks after injection, daily for clinical symptoms, and twice weekly for tumors. If tumors were found or growth to a pre-determined size was observed by the end of the limited period, the animal was euthanatized and the xenograft was removed for further examination. Animals were starved overnight 13 weeks following inoculation, and blood samples were obtained through sinus puncture for hematological examination under ketamine (50 mg/kg, i.p.) anesthesia. Each rat in Groups 4 and 5 received the suspension as a single bolus through a slow subcutaneous injection in the flank area. The injections were given using a sterilized hypodermic syringe and a stainless-steel needle (26 G). The dosage given to each individual rat was 10 mL/kg body weight, which was adjusted based on the rat’s body weight on the day of treatment. 

### 2.8. Histopathological Studies

The animals were sedated with ketamine (50 mg/kg, i.p.) on days 7 and 14, and the whole specimen was removed, along with a thin margin of healthy skin around it from chosen animals. The specimens were preserved for 24 h in buffered 4% paraformaldehyde before carrying out the histopathological analysis. The tissues were mounted on glass slides and stained with hematoxylin and eosin and Masson’s trichrome staining for gross histological assessment and collagen quantification. Using the collected images, the fraction of collagen fibers in the predefined region was calculated.

### 2.9. Immunological Studies

The specimens were embedded in paraffin and dissected into 5 µm thick sections after being fixed in a 10% buffered formalin solution. The sections were deparaffinized and rehydrated with decreasing degrees of alcohol to identify CD68+ macrophages. Following this, they were treated with 3% hydrogen peroxide for 10 min to inhibit the endogenous peroxide activity. Following antigen extraction, an anti-CD68 primary antibody was used. Diaminobenzidine (DAB) was used to detect the bound antibodies. Finally, the slides were mounted and cover slipped after being counterstained with hematoxylin. The semi quantitative assessment of the macrophage infiltration was performed using a bright-field light microscope and a digital camera.

For iNOS (polyclonal rabbit anti-human, Santacruz, CA, USA), tissue section slides were treated in a microwave oven for 10 min with a boiling solution of freshly prepared Tris EDTA buffer, pH 9.0. After cooling to room temperature, the tissue sections were blocked for 10 min with normal goat serum at a dilution of 1:100. The sections were incubated with the primary antibodies in a moist chamber at 4 °C overnight. Each iNOS-specific primary antibody was used at a dilution of 1:100. The slides were then rinsed twice in Tris-buffered saline before treatment for 60 min at room temperature with a dilution of 1:100 goat anti-rabbit horseradish peroxidase (HRP) conjugated secondary antibody.

Polyclonal goat anti-human COX-2 antibody (1:25) (Santacruz, CA, USA) and rabbit anti-goat antibody (HRP-conjugated) (1:100) were used as the primary and secondary antibodies for COX-2 staining. The immunohistochemical reaction was visualized by developing the slides in 3,3′ diaminobenzidine tetra hydrochloride (Vector LaboratoriesInC, Newark, CA, USA) and counterstaining with Mayer’s hematoxylin. After that, the tissue sections were dehydrated, cleared, and mounted. The experiment was carried out three times.

Both the iNOS and COX-2 sections were examined with a Nikon Eclipse 800 microscope (Nikon Corporation, Japan) at a magnification of 200×. The mean average score was used for the heterogeneity analysis. The slides were reviewed at random in order to minimize bias. A consultant histopathologic scored staining by evaluating both the percentage of stained cells and the intensity of the stain within five representative regions of each specimen. Cytoplasmic staining was used to assess the expression.

### 2.10. Estimation of MLD, MTD, and LD_50_

The Litchfield and Wilcoxon technique was used to determine the median lethal dose (LD_50_) value with fiducial at a 95% confidence level (1949). The minimum lethal dosage (MLD) and maximum tolerated dose (MTD) were determined by routine protocols.

### 2.11. Statistical Analysis

The homogeneity of the bodyweight data from different groups was tested using Bartlett’s test and the data were converted using suitable transformations, as needed. The homogeneity of the intra-group variances was studied by one-way ANOVA [13]. Dunnett’s pairwise comparison of the treatment and control group means [14] (Dunnett, 1985) was performed on an individual basis. The variance was evaluated at a 5% level of significance.

## 3. Results

### 3.1. In Vitro Studies

The current study confirmed that the cells derived from *decidua basalis* possess a high plasticity and have the potential to differentiate into several other cell types. Flow cytometric assessment proved that the cells are identical to MSCs. The toxicity assay using Wistar albino rats established the immunological fulfilment of the cells. This experiment successfully identified fibroblast-like mesenchymal stromal cells. These cells were later identified and successfully developed into a cell count of 4.74 × 10^9^ in 9–11 days using a seeding density of 3000 cells/cm^2^. The cultures were found to be highly sterile and free of any aerobic, anaerobic, or fungal contamination when the supernatant was inspected on a regular basis in accordance with previous study reports [15,16]. Endotoxin levels in the final product were less than 0.2 EU/mL.

### 3.2. In Vivo Studies

#### Dosing Formulations and Cell Count

DB-MSCs were subjected to the production of dosing suspensions for individual batches, maintaining a final viable cell concentration of 1 × 10^6^ MSCs/mL with an acceptable variance of 20%. Similarly, the viable cell count of each experimental batch was found to fall well within the required values and adequate standards.

### 3.3. Clinical Signs and Mortality

The day of dosing and regularly thereafter until the 14th day, through frequent clinical examinations at intervals of 30 min, 1 h, 2 h, and 4 h after intravenous/subcutaneous administration, did not reveal any unusual clinical features or preclinical mortalities between the treated rats (Table 1).

### 3.4. Body Weight

The treated animals were not adversely affected during the entire period of investigation (14 days). Table 2 shows that irrespective of gender, the rats injected with MSCs at 10 × 10^6^ MSCs/kg body weight gained mass continuously until they were euthanized on the 15th day. The body weight of the animals was measured on day zero (one day before treatment), day 7, and day 15 just before sacrifice.

### 3.5. Complete Blood Cell Count and Differential Count 

Blood analysis variables were assessed for RBC count, hemoglobin (Hgb) concentration, Hct (instrument-derived), MCV, MCHC, MCH, total WBC count, platelet count, and mean platelet volume (MPV). The automated analyzer used the principal of electrical impedance and a patented focused flow system to measure the cell counts and size. Hgb is measured spectrophotometrically using the cyanmethemoglobin method. The parameters of RBC count, Hgb concentration, and MCV were used to calculate the Hct, MCHC, and MCH. 

### 3.6. Necropsy and Histopathology

The necropsical diagnosis and histopathological studies of the animals injected through both the intravenous and subcutaneous route with DBMSCs did not find any abnormalities in the tissues and organs at the end of the study.

### 3.7. Acute Toxicity and Safety Study

**Figure 1:**
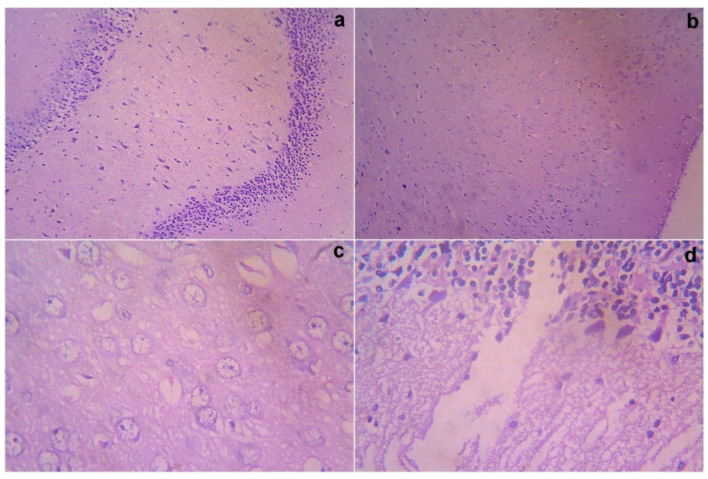
Gross histopathological examination of the brain following the infusion of mesenchymal stem cells obtained from *decidua basalis* in Wistar albino rats to assess the impact of safety, efficacy, and acute toxicity. Representative images of the brain section stand for (**a**) 10× showing a normal hippocampus with mild gliosis, (**b**) 10× showing a normal cerebral cortex, (**c**) 40× showing a hippocampus, and (**d**) 40× showing a normal molecular purkinje cell layer, respectively.

**Figure 2:**
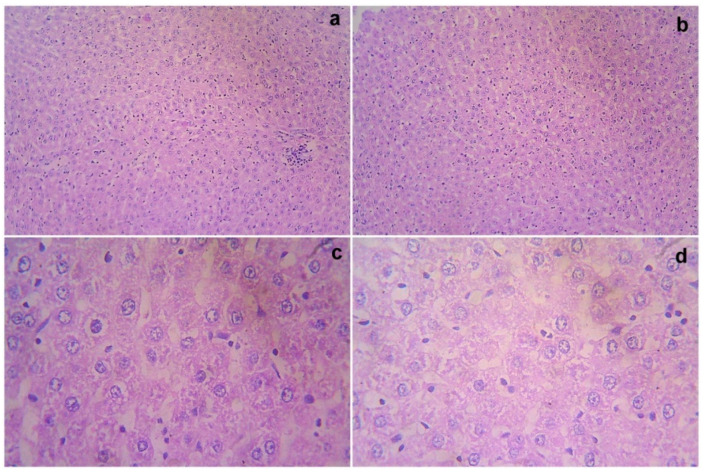
Gross histopathological examination of the liver following the infusion of mesenchymal stem cells obtained from *decidua basalis* in Wistar albino rats to assess the impact of safety, efficacy, and acute toxicity. Representative images of the liver section stand for (**a**) 10× showing central vein and sinusoidal dilatation, (**b**) 10× showing lobular architecture, (**c**) 40× showing focal necrosis, and (**d**) 40× showing cytoplasmic vacuolation and necrosis, respectively.

**Figure 3:**
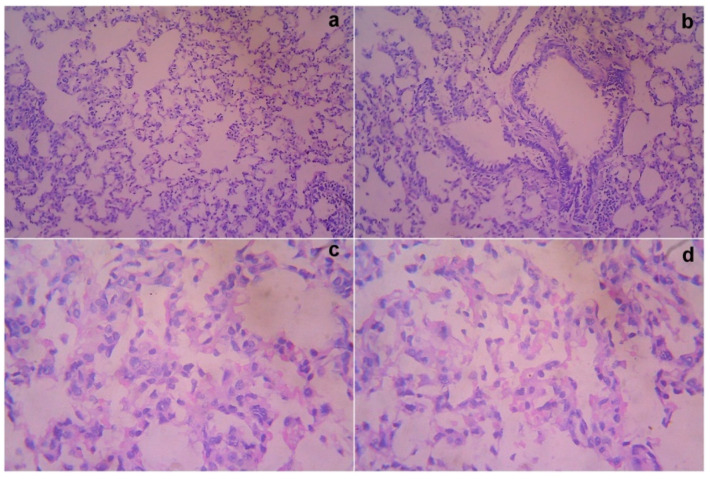
Gross histopathological examination of the lung following the infusion of mesenchymal stem cells obtained from *decidua basalis* in Wistar albino rats to assess the impact of safety, efficacy, and acute toxicity. Representative images of the lung section stand for (**a**) 10× showing alveoli dilatation, (**b**) 10× showing normal bronchi and peribronchiole, (**c**) 40× showing mild dilatation of alveoli, and (**d**) 40× showing destructed architecture, respectively.

**Figure 4:**
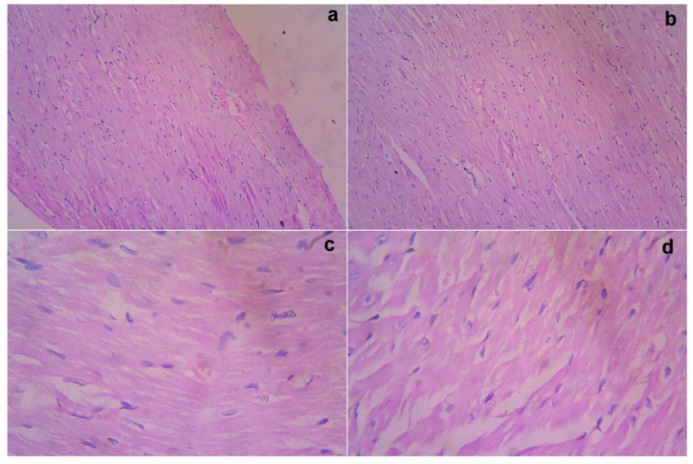
Gross histopathological examination of the heart following the infusion of mesenchymal stem cells obtained from *decidua basalis* in Wistar albino rats to assess the impact of safety, efficacy, and acute toxicity. Representative images of the heart section stand for (**a**) 10× showing the congregation of blood vessels, (**b**) 10× showing myocardium with myocytes, (**c**) 40× showing myocardium with myocytes showing mild edema, and (**d**) 40× showing the myocardium with myocytes showing mild edema, respectively.

**Figure 5:**
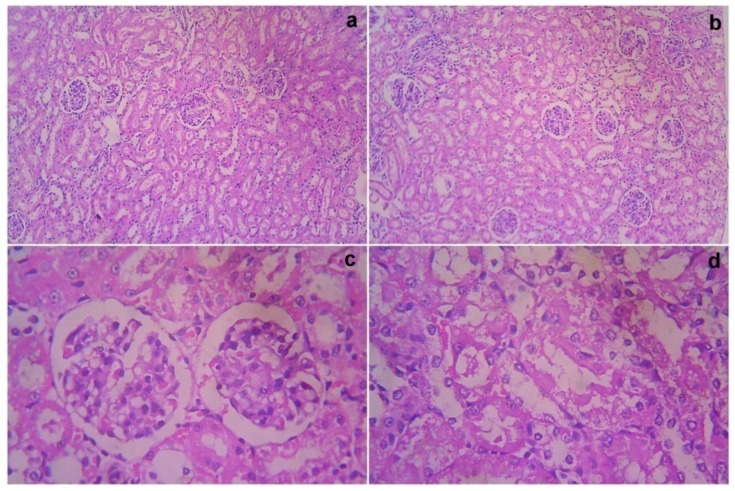
Gross histopathological examination of the kidney following the infusion of mesenchymal stem cells obtained from *decidua basalis* in Wistar albino rats to assess the impact of safety, efficacy, and acute toxicity. Representative images of the kidney section stand for (**a**) 10× showing the kidney with both the cortex and medulla, (**b**) 10× showing normal glomeruli and tubules with interstitial inflammation, (**c**) 40× showing normal glomeruli and tubules showing focal mild epithelial loss, and (**d**) 40× showing tubular epithelial thinning, respectively.

The treated rats (both the genders) did not show any abnormality in the brain, liver, heart, kidney, or lung sections (Figure 1, Figure 2, Figure 3, Figure 4 and Figure 5). Moreover, no significant toxicity was observed in the treated rats during the observation period of 4 to 91 days (Table 3).

### 3.8. Immunological Studies

**Figure 6:**
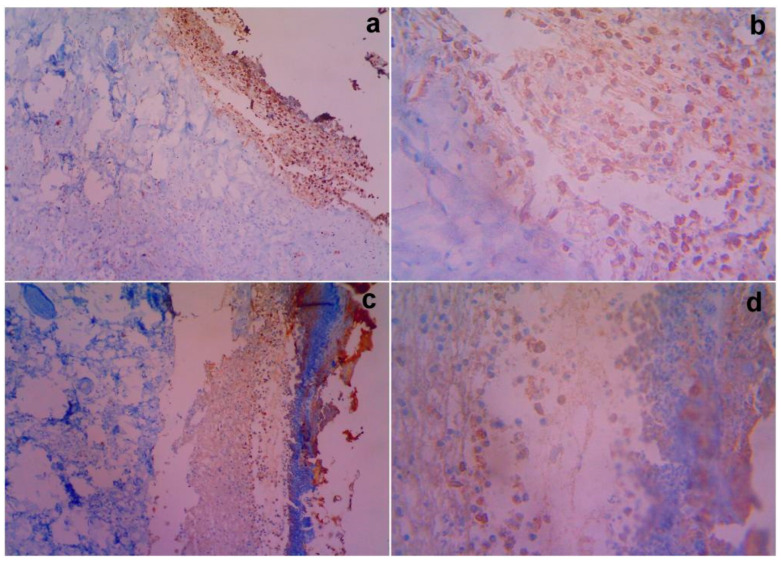
Gross histopathological examination of the different post excision for COX-2 nuclear and cytoplasmic immunoreactive positivity with treated rats on day 14. Representative images of the COX-2 nuclear and cytoplasmic section stand for (**a**) 10× showing magnification of the control showing 3(+) immunoreactivity compared with Group II (**c**) showing 2(+) immunoreactivity for MSCs, (**b**) 40× showing magnification of 3(+) immunoreactivity by the vehicle control, and (**d**) 40× showing magnification of 2(+) immunoreactivity by the test article, respectively.

**Figure 7:**
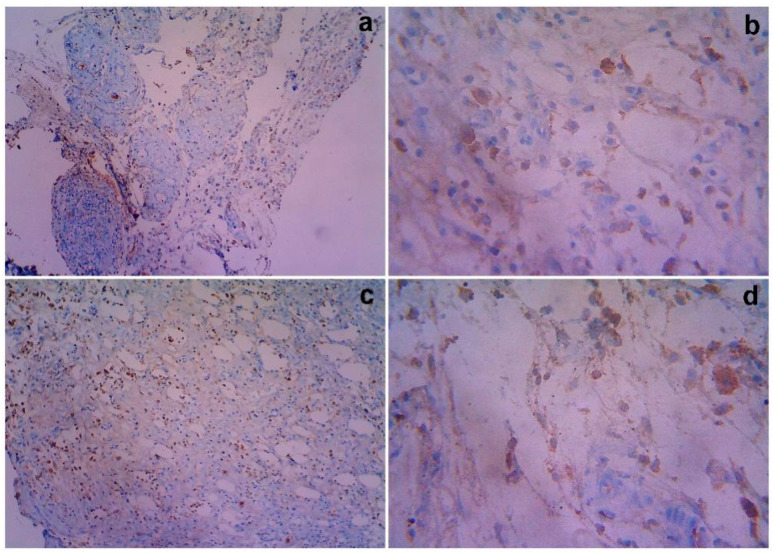
Gross histopathological examination of the different post excisions for iNOS nuclear and cytoplasmic immuno reactive positivity with treated rats on day 14. Representative images of the iNOS nuclear and cytoplasmic section stand for (**a**) 10× showing iNOS for the untreated control cytoplasmic positivity (1+) and immunoreactivity (1+), (**b**). 40× shows granulation tissue shows 1+ respectively. iNOS for MSCs treated group shows Cytoplasmic positivity (2+) and Immunoreactive (2+): (**c**). 10× shows immuno reactive 2+, (**d**). 40× shows immuno reactive 2+, respectively.

The data regarding the expression of pro-inflammatory protein cyclooxygenase-2 (COX-2) immunoreaction, nuclear and cytoplasmic reactions on days 14 and 21 are supplied in Table 4. Higher nuclear and cytoplasmic reactions were observed in the Group 2 (G2) animals on day 14, which were reduced on day 21. The expression of pro-inflammation in G2 was much greater and new blood vessel formation was higher. The untreated control (G1) showed higher inflammation and unperturbed recovery compared with the treated animals (G2) (Figure 6). The data pertaining to the expressions for the anti-inflammatory response of iNOS nuclear and cytoplasmic positivity on 14 and 21 are shown in Table 4. In Group 3, lower nuclear and cytoplasmic reactions (1+) were observed on day 14. However, in case of Group IV, the results showed higher nuclear and cytoplasmic reactions (2+) on day 14 (Figure 7). 

### 3.9. Calculation of MTD, MLD and LD_50_

As neither death nor adverse effects were witnessed among the treated animals, the MLD, MTD, and LD_50_ of DBMSCs injected through intravenous and subcutaneous routes were not determined under the dosage conditions. Thus, it can be inferred that the MLD, MTD, and LD_50_ of DBMSCs should be >10 × l0^6^ MSCs/kg body weight.

### 3.10. Statistical Analysis

After intravenous injection (G2 and G3), the body weight of the treatment group rats did not vary significantly compared with that of the control group animals (*p* >0.05), with the exception of a few lower values in G2 (Table 2), which had statistical significance. This apparent lowering was comparable to the normal weight gain pattern of Wistar albino rats. Given the small sample size (five per gender), the statistical finding was more likely to be coincidental.

After subcutaneous injection (G4 and G5), the change in the body weight for the treatment group rats was not of statistical significance compared with the control group (*p* >0.05), with the exception of a few male rats in both of the groups, in which case the weight gain was slightly lower after the first week. However, this anomaly can be substantiated because of the varying stocking density of the animals.

## 4. Discussion

The present study shows that the cells obtained from *decidua basalis* looked similar to fibroblast-like cells, showed mesenchymal features such as plastic adherence and tri-lineage differentiation, expressed phenotypically comparable markers, and could be scaled up for therapeutic use. However, it is critical to determine the functional significance of these cells in terms of their homing capacity, as well as to optimize the appropriate route of delivery based on the pathophysiology of the patient. The goal of the animal experiment is to assess the clinical toxicity associated with therapeutic applications and the best method of administration in order to address the concerns. The test product (MSCs) was given to five groups of Wistar albino rats of both sexes in a single dosage. Subcutaneous and intravenous injections of MSCs were used to administer MSCs to rats, as both methods assure systemic dispersion and could be used as a therapeutic intervention. The rats were monitored for 14 days to check for symptoms of toxicity and fatalities. In the absence of any hazardous reaction, the timeframe was not prolonged. The trial was conducted at a limiting dosage of 10 × 10^6^ MSCs/kg body weight because it provides a tolerable margin for the proposed therapeutic use [17,18,19,20,21,22,23]. When DBMSCs were infused into Wistar Albino rats at an acute dose, intravenously and subcutaneously at a drug concentration of 10 × 10^6^ MSCs/kg body mass, the treated rats were not subjected to any fatalities, unusual clinical manifestations, or body weight gain during the monitoring period that followed the intervention. Furthermore, the treated rats did not develop any gross pathological changes in their tissues or organs. The complete blood cell count and differential count of the treated male and female rats during the 21-day observation period of this study were found on the blood sample to not show any abnormal effects due to the administration of mesenchymal stem cells. The MLD, MTD, and LD_50_, which are quantitative measurements of the acute intravenous and subcutaneous toxicity of DBMSCs in Wistar Albino rats, were calculated to be larger than 10 × l0^6^ MSCs/kg body weight, or 10 times the maximum human therapeutic dosage. Thus, the MLD, MTD, and LD_50_ of DBMSCs were not determined as the observations did not necessitate it.

Angiogenesis and regeneration mechanisms are the result of the triggering of various signaling processes. Angiogenesis in rat models is mediated by two enzymatic pathways: the cyclooxygenase (COX) pathway and the nitric oxide synthase (NOS) pathway. The isoform COX-1 is essential for maintaining prostaglandin levels, which takes care of various indispensable biological processes. Similarly, NO plays an inevitable role in immune responses [24,25]. Isoform inducible NOS (iNOS) can be expressed in response to pro-inflammatory agents. Previous research has shown that iNOS plays a significant role in retinal angiogenesis. Furthermore, it has been reported that iNOS and COX-2 can regulate each other’s activity [26].

The expression of the pro-inflammatory protein COX-2 in animal models, its relationship with angiogenesis, and its clinic pathological correlation were investigated. One major etiology for mesenchymal stem cell homing is cyclooxygenase-2 (COX-2). Several pro-inflammatory gene products have been identified that play an important role in apoptosis, proliferation, angiogenesis, invasion, and metastasis [27,28]. Among these gene products, COX-2 is closely related with angiogenesis and mesenchymal stem cell homing. In the tissue samples, COX-2 and iNOS inflammatory mediators were measured. Only the plasma membrane of malignant epithelial cells was considered COX-2 positive in the COX-2 expression. The staining region was scored as follows when viewed under magnification. The stain’s intensity was measured on the following scale: 0 indicates no staining, 1 indicates mild staining, 2 indicates moderate staining, and 3 indicates intense staining. The staining area was assessed as follows: 0, no stained cells in any microscopic field; 1, less than 25% of cells stained positively; 2, between 25% and 50% of cells stained positively; 3, between 50% and 75% of cells stained positively; and 4, more than 75% of cells stained positively. According to Brennan et al. [29], the sum of the staining area and intensity was used for the statistical analysis. For example, if the intensity of the stain is 3 and the area of staining is 4, the sum is 3 plus 4, which equals 7. The minimum and maximum scores in this analysis were zero and seven.

The results of the COX-2 expression revealed a higher nuclear and cytoplasmic reaction on day 14, which was reduced on day 21 (Group II). This indicates that inflammation was reduced dramatically as a result of the development of new blood vessels and regeneration. The expression of pro-inflammation in the group (G2) was much greater and angiogenesis was higher. The untreated control (G1) showed higher inflammation and unperturbed recovery compared with the treated animals (G2). Hence, this study revealed COX-2 expression in the treated animals, and therefore the expression of COX-2 inflammatory biomarkers proved the progression of the regeneration and formation of new blood vessels. The expression for the anti-inflammatory response of iNOS nuclear and cytoplasmic positivity for days 14 and 21 suggest a lower nuclear and cytoplasmic reaction (1+), as observed on day 14. This indicates that the inflammatory response was suppressed and regeneration was induced in Group 3. Similarly, higher nuclear and cytoplasmic reactions (2+) were observed among the Group 4 animals, indicating a lesser anti-inflammatory response compared with the vehicle control groups. These results prove that DBMSCs expanded under clinical scale level and iNOS was expressed in response to pro-inflammation and COX-2 was induced in response to the homing of mesenchymal stem cells.

## 5. Conclusions

The focus of this research was to determine the acute toxicity of *decidua*-*basalis*-derived mesenchymal stem cells in Wistar albino rats through intravenous and subcutaneous routes, as well as to determine their propensity for side effects, MLD, MTD, and LD_50_. A broad range of pre-clinical and clinical trials have recommended using adult MSCs for cell-based reparative therapeutic approaches. Among them, *decidua basalis* is of paramount interest due to its multitude of advantages. In this investigation, DBMSCs were isolated, thoroughly characterized, and studied further for scaling up. In addition, harmful effects of these cells were assessed when delivered in vivo and the relevant clinical parameters were investigated. It was found that these cells had morphological and phenotypic features that are comparable to MSCs. In the animal studies, there was no mortality, aberrant clinical indications, or significant pathological alterations in the animals in the research. The results of the animal toxicity investigation, together with the attempt to rapidly expand these cells to satisfy the significant clinical needs, suggest that they are an area of promise for clinical therapeutic application. Furthermore, randomized, controlled, multicenter clinical studies are needed to explore the best circumstances for MSC treatment. These cells will play a key role in the treatment of numerous illnesses such as Alzheimer’s disease, Parkinson’s, spinal cord injury, stroke, cancer, cerebral palsy, and Battens disease, as well as other immune-related and neurodegenerative diseases that currently have no viable treatment methods.

## Figures and Tables

**Table 1:** Summary of Mortality.

Groups	Drug Treatment	Incidence of Mortality					
		Male Rats		Female Rats		Male and Female Rats	
		Absolute	% Mortality	Absolute	% Mortality	Absolute	% Mortality
I Vehicle control	10 mL/kg Sodium Chloride Inj.	0/5	0	0/5	0	0/10	0
II Test article (Intravenous)	MSCs 10 × 10^6^ MSCs/kg	0/5	0	0/5	0	0/10	0
III Test article (Subcutaneous)	MSCs 10 × 10^6^ MSCs/kg	0/5	0	0/5	0	0/10	0

Absolute mortality is presented as the number of animals that died/numbers treated.

**Table 2:** Summary of male and female rat body weights after intravenous and subcutaneous injection.

Mode	Body Weights (g)		Male Rats					Female Rats				
	Groups	Observation	Day 1	Day 7	Change (1–7)	Day 15	Change (1–15)	Day 1	Day 7	Change (1–7)	Day 15	Change (1–15)
Control (10 mL saline/kg)	**G1**	Mean	181.5	187.6	6.1	193.8	12.3	177.2	180.7	3.5	184.9	7.7
		± S. D.	2.1	1.9	3.1	3.4	5.1	1.4	2.8	2.5	3	2.7
		n	5	5	5	5	5	5	5	5	5	5
MSCs Treated rats (Test group) 10 × 10⁶ MSCs/kg	**G2**	Mean	180.2	184.1	3.9	186.5 ^S−^	6.3	175.3	178.3	3	184.3	9
		± S. D.	0.2	1	0.8	0.4	0.5	1.7	2.9	1.7	3.2	2.5
		n	5	5	5	5	5	5	5	5	5	5
	**G3**	Mean	183.2	186.8	3.6	191.3	8.1	178.7	181.6	2.9	186.1	7.4
		± S. D.	1.2	2.2	1.2	3.4	2.2	1.3	1	1.9	2.6	3.7
		n	5	5	5	5	5	5	5	5	5	5
	**G4**	Mean	182.6	186.2	3.6	188.5	5.9 ^S−^	174.8	176.1	1.3	181.3	6.5
		± S. D.	1.1	0.6	0.7	1.7	1.7	2	2.4	1.5	2.7	1.3
		n	5	5	5	5	5	5	5	5	5	5
	**G5**	Mean	181.5	183.1 ^S−^	1.6	187.8	6.3	175.2	177.4	2.2	181.8	6.6
		± S. D.	1.2	0.3	1.1	2.2	1.1	2.2	3.2	1	2.2	0.1
		n	5	5	5	5	5	5	5	5	5	5

Values of treatment group mice do not differ significantly from those of the control group at 5% level significance. ^S−^: Values significantly lower than those of the control group, with *p* < 0.05.

**Table 3:** Summary of tumorigenicity.

Group	Dose 10 × 10⁶ MSCs/kg Body Weight	Incidence of Tumorigenicity					
		Male		Female		Male and Female (Pooled)	
		Absolute	%	Absolute	%	Absolute	%
G1 Vehicle control	10 mL/kg Sodium Chloride Inj.	0/5	0	0/5	0	0/10	0
G2	10	0/5	0	0/5	0	0/10	0
G3	10	0/5	0	0/5	0	0/10	0
G4	10	0/5	0	0/5	0	0/10	0
G5	10	0/5	0	0/5	0	0/10	0

Absolute tumorigenicity is presented as number of animals died/numbers treated.

**Table 4:** Summary of mortality.

Groups	Drug Treatment	Incidence of Mortality					
		Male Rat		Female Rat		Male and Female Rat	
		Absolute	% Mortality	Absolute	% Mortality	Absolute	% Mortality
I Vehicle control	10 mL/kg Sodium Chloride Inj.	0/5	0	0/5	0	0/10	0
II Test article (Intravenous)	MSCs 10 × 10^6^ MSCs/kg	0/5	0	0/5	0	0/10	0
III Test article (Subcutaneous)	MSCs 10 × 10^6^ MSCs/kg	0/5	0	0/5	0	0/10	0

Absolute mortality is presented as the number of animals that died/numbers treated.

## Data Availability

The datasets and all other information are available with corresponding author and data will send on mail request.
